# Supplementary material for: OATD-02 Validates the Benefits of Pharmacological Inhibition of Arginase 1 and 2 in Cancer
Source: Cancers (Basel). 2022 Aug 17;14(16):3967. doi: 10.3390/cancers14163967 (PMC9406419; doi:10.3390/cancers14163967)
Supplement: Supplementary file 1 [file cancers-14-03967-s001.zip › cancers-1826000-supplementary.pdf]

Supplementary Materials

Western blots for K562 cell lysate showing ARG1 and ARG2 bands with densitometry readings were shown in Figure S1.

Detection of **ARG1** (above) and **ARG2** (below)	Detection of β-tubulin (stripped membranes)
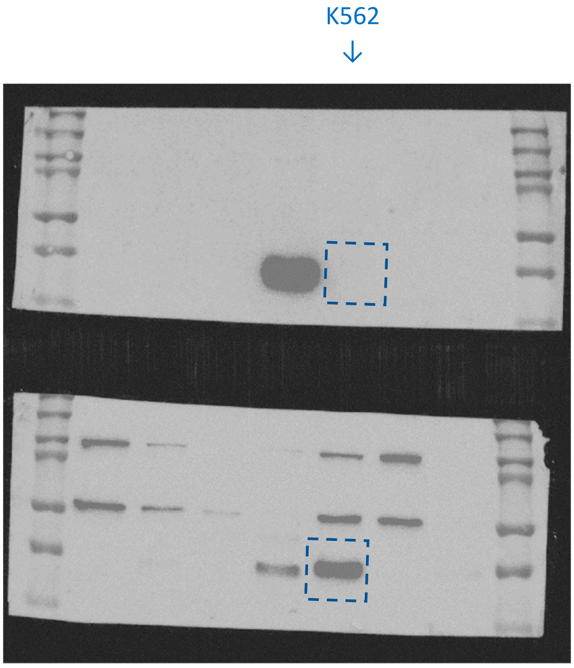	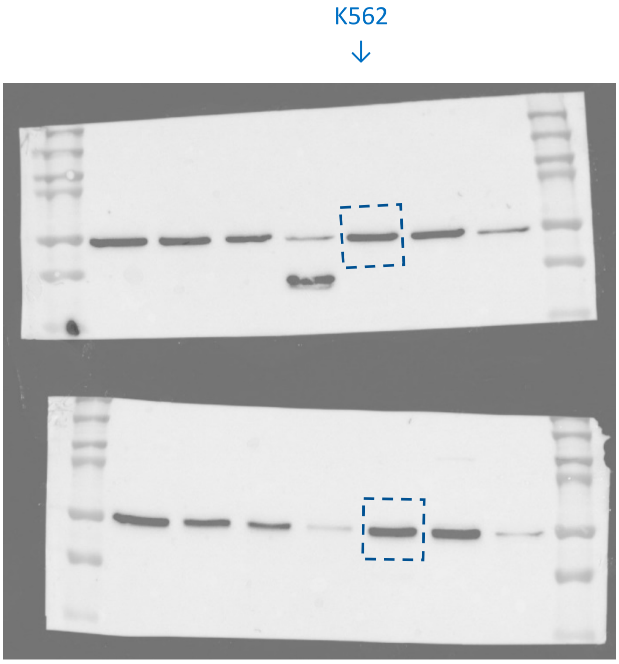
Densitometry readings	
**ARG1** = 221<br>**ARG2** = 34443	β-tubulin (**ARG1**) = 14281<br>β-tubulin (**ARG2**) = 14564
**ARG1**/β-tubulin = 0.016<br>**ARG2**/β-tubulin = 2.365	

Figure S1. ARG1 and ARG2 expression (left panel) was confirmed in K562 cell lysate by SDS-PAGE and western blotting with specific antibodies; after stripping, detection of β-tubulin (right panel) was performed.
